# Methimazole-induced cholestatic hepatitis: two cases report and literature review

**DOI:** 10.18632/oncotarget.6144

**Published:** 2015-10-19

**Authors:** Hai Zou, Lie Jin, Li-Ren Wang, Martin Braddock, Wen-Wei Cai, Ming-Hua Zheng

**Affiliations:** ^1^ Department of Emergency, Zhejiang Provincial People's Hospital, Hangzhou, China; ^2^ Department of Nephrology, Lishui Hospital Affiliated to Zhejiang University, Lishui, China; ^3^ Department of Infection and Liver Diseases, Liver Research Center, The First Affiliated Hospital of Wenzhou Medical University, Wenzhou, China; ^4^ School of the First Clinical Medical Sciences, Wenzhou Medical University, Wenzhou, China; ^5^ Global Medicines Development, AstraZeneca R&D, Alderley Park, United Kingdom; ^6^ Institute of Hepatology, Wenzhou Medical University, Wenzhou, China

**Keywords:** cholestatic hepatitis, methimazole, adverse effect

## Abstract

Methimazole is commonly prescribed for patients who are thyrotoxic. Cholestatic hepatitis is a rare but serious adverse event which may be associated with interventional therapy. In this case report, we present two Chinese women with cholestatic jaundice due to methimazole treatment. Both patients had a history of hyperthyroidism; initial laboratory studies of liver function were normal and cholestatic hepatitis occurred after treatment with methimazole. Concomitant liver disease, such as viral hepatitis (A, B, C, D, E), autoimmune hepatitis, primary biliary cirrhosis and calculus of bile duct, were excluded. Liver enzyme levels in both patients returned to normal after stopping methimazole therapy and taking hepatoprotective drugs. It is essential that patients are informed about the earliest symptoms of serious adverse effects of antithyroid drugs, such as hepatic toxicity, and that they are advised to stop taking the drug immediately and contact their physician if such symptoms occur.

## INTRODUCTION

Methimazole is commonly prescribed for patients who are thyrotoxic. Cholestatic hepatitis is a rare but serious adverse event and although the mechanism of the event is not clear, some studies attribute the cause to an allergic reaction. In this case report, we present two Chinese women with cholestatic jaundice due to methimazole treatment.

## CASE REPORT

### Case 1

A 50 year old woman presented with a change in urine color and severe pruritus and was admitted to our hospital on February 14, 2014. Hyperthyroidism had been diagnosed based on an elevated plasma free thyroxine (FT4) level of 96.1 (normal, 12.0–22.0 pmol/l), free triiodothyronine (FT3) 22.7 (normal, 3.1–6.8 pmol/l), and thyrotropin level of < 0.005 (normal, 0.270–4.200 mIU/l), as well as diarrhea and weight loss 8 days previous (Table 1). The initial laboratory studies showed normal liver function and a complete blood count. Treatment started with 10 mg of methimazole added onto 10 mg of propranolol, three times daily. 8 days after starting methimazole, the patient began to show severe pruritus and a change in urine color. She did not have abdominal pain, nausea, fever and signs of heart failure, had no history of alcohol drinking or liver disease and also reported no history of using any other drugs except methimazole and propranolol. At admission, physical examination revealed that her blood pressure was 120/76 mmHg and her pulse rate was 88 beats per minute. She had severe icterus of the sclerae and skin. There were no signs of chronic liver disease. Her thyroid was diffusely enlarged. Respiratory and cardiovascular examinations were normal, abdominal examination was unremarkable and the liver and spleen were not palpable. Laboratory investigations revealed abnormally high serum FT4 (56.33 pmol/l) and FT3 (11.86 pmol/l) and low TSH (< 0.005 mIU/l). Hepatic function tests revealed total bilirubin 105.86 μmol/l (normal, 3.40–25.00 μmol/l), direct bilirubin 54.21 μmol/l (normal, 0.00–8.00 μmol/l), alkaline phosphatase (ALP) 200 U/L(normal, 34–104 U/L), gamma-glutamyl transferase (GGT) 104 U/L (normal, 0–60 U/L), aspartate aminotransferase (AST) 17 U/L (normal, 0–50 U/L), and alanine aminotransferase (ALT) 120 U/L (normal, 5–64 U/L); Albumin, prothrombin time, partial thromboplastin time values and blood cell count were within normal limits. Serology for hepatitis viruses (A, B, C, D, E), Epstein-Barr and cytomegalovirus (CMV) were all negative. Auto-antibody profiles for primary biliary cirrhosis and autoimmune hepatitis including ANA (anti nuclear antibody), ASMA (anti smooth muscle antibody), LKM (liver kidney microsomal antibody), AMA (anti mitochondrial antibody), mpo-ANCA (myeloperoxidase-antineutrophil cytoplasmic antibody) and anti-dsDNA were all negative. An ultrasonogram of the abdomen showed unremarkable changes of the liver, pancreas, and spleen, without any evidence of biliary dilation. In the absence of any mechanical obstruction in the common bile duct or other obvious causes of hepatic injury, and because of the temporal relationship between initiation of methimazole and onset of cholestasis, a diagnosis of drug-induced liver disease was suspected. Treatment with methimazole was stopped and propranolol therapy was resumed for symptomatic relief. The patient commenced therapy with magnesium isoglycyrrhizinate and other hepatoprotective drugs. Liver enzyme values were closely monitored during hospitalization, and the symptoms gradually resolved. Serum levels of liver enzymes (ALT and AST) and bilirubin were nearly normal at 4 weeks after the discontinuance of methimazole therapy. The patient refused thyroidectomy or radioactive iodine therapy. Propylthiouracil (PTU) was given for hyperthyroidism and liver function was monitored continuously during the switch to PTU therapy. The patient is still taking PTU 50 mg per day, with normal thyroid and hepatic function.

**Table 1:** The laboratory values of two cases

Laboratory Variable	Case 1	Case 2	Normal range
FT4 (pmol/l)	56.33	24.48	0.61–1.12
FT3 (pmol/l)	11.86	5.62	2.50–3.90
TSH (mIU/l)	< 0.005	0.008	0.30–4.80
Total bilirubin (μmol/l)	105.86	268.87	3.40–25.00
Direct bilirubin (μmol/l)	54.21	116.80	0.00–8.00
ALP (U/L)	200	353	34–104
GGT (U/L)	104	255	0–60
AST (U/L)	17	105	0–50
ALT (U/L)	120	135	5–64

FT4: free thyroxine; FT3: free triiodothyronine; TSH: thyroid stimulating hormone; ALP: alkaline phosphatase; GGT: gamma-glutamyl transferase; AST: aspartate aminotransferase; ALT: alanine aminotransferase;

### Case 2

A 42 year old woman was referred to our ward because of decreased appetite and severe pruritus and presented on July 9, 2014. Hyperthyroidism was diagnosed based on an elevated FT4 level of 32.84 pmol/l, FT3 11.64 pmol/l, and thyrotropin level of 0.005 mIU/l, as well as palpitations and hand tremors 1 month prior to admission to hospital (Table 1). The initial laboratory studies showed normal liver function and a complete blood count. Methimazole therapy at 30 mg/day was started. Two weeks after using methimazole, the patient began to have pruritus, which she considered as a drug allergy. She decreased the dose of the methimazole to 15 mg/day and continued taking her medication until her appetite decreased and she presented with severe pruritus after 27 days taking methimazole. The patient had no medical history except hyperthyroidism and had never consumed alcohol. She had severe icterus of the sclerae and skin and her thyroid was diffusely enlarged. Other physical examinations were considered normal. Laboratory investigations showed FT3 5.62 pmol/l and FT4 24.48 pmol/l, and TSH 0.008 mIU/l. Hepatic function tests revealed total bilirubin 268.87 umol/L, direct bilirubin 116.80 umol/L, ALP 353 U/L, GGT 255 U/L, AST 105 U/L, and ALT 135 U/L. Concomitant liver diseases, such as viral hepatitis (A, B, C, D, E), autoimmune hepatitis, and primary biliary cirrhosis, were excluded by serological analyses. A diagnosis of methimazole-induced cholestasis was made and methimazole therapy was stopped and hepatoprotective drugs were given as described in Case 1. However, after two weeks of admission, the patient had progressive worsening of nausea, anorexia, itching, and jaundice. Highest values recorded were: total bilirubin 348.20 umol/L, direct bilirubin 262.12 umol/L. Methylprednisolone was added by phleboclysis and 6 days later, bilirubin values fell significantly (total bilirubin 183.21 umol/L, direct bilirubin 157.90 umol/L). Oral methylprednisolone was subsequently prescribed, which followed a tapering regime and was discontinued after 16 weeks, resulting in a normal liver function. For treatment of hyperthyroidism, the patient would be a candidate to receive radioactive iodine ablation of the thyroid gland.

## DISCUSSION

Methimazole is a widely used and generally well-tolerated antithyroid agent. Adverse reactions to antithyroid drugs occur in less than 10% of patients being treated for hyperthyroidism. Most of these side effects are mild, and can be self-resolved after discontinuing the drugs. Methimazole-induced cholestasis is extremely rare, and fewer than 30 cases are reported in the literature. Women appear more prone to methimazole-induced cholestasis [1] and this finding most likely reflects the predominance of hyperthyroidism in the female sex. A latent period between drug exposure and the development of jaundice ranges from 2 days to 3 months and the reaction to the drug is dose-independent. The outcome of methimazole-induced cholestasis is generally benign, with self-resolution of symptoms and normalization of bilirubin levels and other liver function values resulting 5 days to 6 months after stopping methimazole therapy. In some rare cases, however, methimazole therapy may cause death [2].

In these two cases, cholestatic jaundice was most likely caused by methimazole for the following reasons. First, there was a clear temporal relationship between the initiation of methimazole and the development of jaundice and the patient's clinical status and laboratory findings have improved with appropriate treatment since hospital admission. Secondly, the patients appear to have no past history and no recent risk factors associated with liver disease and concomitant liver disease was excluded by serological tests. Thirdly, it is unlikely that the severe cholestasis presented was related to hyperthyroidism. In our cases, thyroid hormone values had already improved, while recorded plasma bilirubin levels were very high. Liver biopsy and rechallenge with methimazole was not performed in the present cases because both procedures are not without risks and significant discomfort to the patient.

The mechanism of methimazole-induced cholestasis has not been fully elucidated. Possible mechanisms include the following: (1) relative hypoxia of the portal system may bring about liver cell degeneration and necrosis; (2) individual bile metabolic disorders, such as Gilbert syndrome and Dubin-Johnson syndrome, become manifest after medication; (3) several active substances produced during the process of methimazole metabolism may damage the liver organellae and stroma by numerous routes which include, individual differences in hepatic cytochrome P450 enzymes [3]; (4) an allergic reaction [4]. However, a number of observations suggest that it is most likely an allergic reaction.

Propylthiouracil (PTU) is another antithyroid drug and cross-allergic reactions may exist between propylthiouracil and methimazole. However, some important differences exist with respect to hepatotoxic effects. The latent period preceding the appearance of symptoms after starting the drug is more extended with propylthiouracil, ranging from 1 day to 15 months [1] and the drug reaction is non-dose-independent. Moreover, The United States Food and Drug Administration (FDA) has added a boxed warning to the label for propylthiouracil, to include information about reports of severe liver injury and acute liver failure, some of which have been fatal, in adult and pediatric patients using this medication. In view of this, the best treatment for hyperthyroidism in patients with cholestatic jaundice due to methimazole is thyroidectomy or radioactive iodine therapy. However, some patients have switched to PTU with a successful outcome [5], as presented in case 1.

Disease of the liver and thyroid are interrelated to each other and are mutually affected and thyroxin will cause disorders of liver circulation when hyperthyroidism develops. Hyperfunction of body catabolism may lead to liver cell hypoxia and dystrophy and result in liver cell injury. In parallel, hyperthyroidism may make the liver more susceptible to injury by anoxic, infection and virulence factors and pharmacological agents. Thyroxin is metabolized in the liver and disorders in the liver reduce the thyroxin inactivation resulting in an increase in thyroxin levels. Methimazole-induced cholestasis will recover quickly after drug withdrawal, however, in cases with severe hepatitis, liver function is usually very poor, and patients cannot normally tolerate antithyroid drugs, radioactive iodine therapy or thyroidectomy. If hyperthyroidism cannot be controlled, it may aggravate hepatitis. Treatment with corticosteroids has been attempted but did not appear to accelerate the recovery process in some cases [6], however, in case 2 and in other patients, corticosteroids successfully improved drug-related cholestatic hepatitis [7]. Although corticosteroid therapy is not the only effective therapy to treat jaundice, it can control hyperthyroidism in the short term.

There are some new findings in this study. First of all, measured liver enzymes including ALT and AST for both cases were highly elevated when comparing with those found in the normal range. Progress of abnormally elevated liver enzymes has proceeded rapidly, and the ALT in case 1 was twice that of the normal range after an eight day period. Lastly, the hepatic function test of the both cases was only rechecked infrequently, resulting in severe elevation of the liver enzymes over time. To avoid this in future, it is recommended that a liver function test of patients receiving methimazole be performed every 2 or 3 days.

In summary, in rare cases within the first few weeks of therapy, methimazole can cause severe and reversible cholestatic jaundice. Physicians and patients should be aware of this rare but serious adverse effect. Meanwhile, it is essential that patients are informed about the earliest symptoms of serious adverse effects of antithyroid drugs, such as those of agranulocytosis and hepatic toxicity, and that they are advised to stop taking the drug immediately and contact their physician if such symptoms occur.

## Abbreviations

FT4: free thyroxine
FT3: free triiodothyronine
TSH: thyroid stimulating hormone
ALP: alkaline phosphatase
GGT: gamma-glutamyl transferase
AST: aspartate aminotransferase
ALT: alanine aminotransferase
CMV: cytomegalovirus
PTU: propylthiouracil
FDA: Food and Drug Administration
